# Transmembrane and Ubiquitin-Like Domain Containing 1 (Tmub1) Regulates Locomotor Activity and Wakefulness in Mice and Interacts with CAMLG

**DOI:** 10.1371/journal.pone.0011261

**Published:** 2010-06-22

**Authors:** Wandong Zhang, Katerina V. Savelieva, Adisak Suwanichkul, Daniel L. Small, Laura L. Kirkpatrick, Nianhua Xu, Thomas H. Lanthorn, Gui-Lan Ye

**Affiliations:** Neuroscience Research, Lexicon Pharmaceuticals Inc., The Woodlands, Texas, United States of America; L'université Pierre et Marie Curie, France

## Abstract

Tmub1 (C7orf21/HOPS) encodes a protein containing a ubiquitin-like domain. Tmub1 is highly expressed in the nervous system. To study its physiological function, we generated mice with Tmub1 deleted by homologous recombination. The knockout mice were grossly normal and viable. In a comprehensive behavioral testing battery, the only knockout phenotype displayed was a strong increase in home cage locomotor activity during the dark phase (subjective day) of the light∶dark (L∶D) cycle. There were no changes in activity during the light period. There were no changes in locomotor activity observed in other assays, e.g. novel open-field. The increase in dark phase locomotor activity persisted during a seven day D∶D (complete darkness) challenge, and remained largely confined to the normally dark period. Telemetric recording in freely moving subjects for one 24 hr L∶D cycle, revealed the same increase in locomotor activity in the dark phase. In addition, EEG analysis showed that the knockout mice exhibited increased waking and decreased NREM & REM times during the dark phase, but the EEG was otherwise normal. Using lacZ as a reporter we found Tmub1 expression prominent in a few brain structures including the thalamus, a region known to drive wakefulness and arousal via its projections to the cortex. We identified calcium modulating cyclophilin ligand CAMLG/CAML as a binding partner by a yeast two-hybrid screen of a brain library. The interaction of Tmub1 and CAMLG was confirmed by co-immunoprecipitation assays in HEK cells. The two proteins were also found to be co-localized to the cytoplasm when expressed in HEK cells. Both Tmub1 and CAMLG have been recently described in the regulation of membrane trafficking of specific receptors. Taken together our results implicate Tmub1 in the regulation of locomotor activity and wakefulness and suggest that Tmub1 binds to and functions together with CAMLG.

## Introduction

Tmub1 encodes for a protein, HOPS/DULP, containing transmembrane domains and a ubiquitin-like domain (UBL) [1], [2]. Ubiquitin can be employed to modify proteins by either single or multiple ubiquitin conjugation to mark them for proteasomal destruction, endocytosis and binding to other proteins [3]–[5]. Ubiquitin-like domain containing proteins are generally divided into two categories: the ubiquitin-like modifiers, such as SUMO, which can conjugate proteins similar to ubiquitin [6]–[8], and the integral UBL domain-containing proteins (UDP) that are not conjugatable. The functions of UDPs are believed to be involved in a wide range of cellular processes. Some UDP proteins function by binding to the 26S proteasome via their UBL domain, whereas others function independent of the proteasome, including roles in receptor trafficking [3], [9], [10]. The UBL containing proteins Plic-1/ubiquilin-1 and GABARAP/ubiquilin-2, both of which also contain a ubiquitin-associated domain (UBA), were shown to regulate GABAA receptor cellular localization and cell surface number [11]–[13]. Tmub1/HOPS was reported to facilitate the recycling of AMPA receptors into synaptic membrane in cultured primary neurons [14]. Tmub1/HOPS was also found overexpressed during liver regeneration [1] and is an essential constituent of centrosome assembly during cell cycles in culture [15]. Like many UBL domain proteins, the physiological and molecular function of Tmub1 is far from being clear. To investigate the functions of Tmub1 *in vivo*, we performed Tmub1 gene deletion in mice, as part of a collaborative effort between Genentech and Lexicon Pharmaceuticals to analyze the function of about 500 secreted and transmembrane proteins. The resulting Tmub1 −/− mice were put through an extensive battery of assays to discover physiological and behavioral phenotypes. We also examined the regional expression in brain by the promoter reporter LacZ, the subcellular localization of protein in HEK cells, and used a yeast two hybrid screen to look for protein partners.

## Results

Tmub1 KO mice appeared normal in terms of weight, length and in a battery of physiological and metabolic assays (data not shown) routinely run on all mice generated with genetic deficiencies at Lexicon Pharmaceuticals [16]. General activity, anxiety, motor coordination, acoustic startle, sensorimotor gating, depressive-like behaviors, learning and memory, and acute and tonic pain sensitivity did not differ between KO mice and WT controls as assessed in the open field, stress-induced hyperthermia, marble burying, inverted screen, pre-pulse inhibition, tail suspension, trace fear conditioning, hot plate and formalin paw assays (Table 1).

**Table 1:** General behavioral characterization of Tmub1 KO mice.

	WT	KO
**Circadian (only females)**		
**First 1 hour habituation**	900±232 (6)	1109±425 (10)
**First 10 hours habituation**	2713±592 (6)	12785±13996 (10)*
**Average light cycle activity**	789±387 (6)	816±929 (10)
**Average dark cycle activity**	2344±882 (6)	15531±22340 (10) #
**Open Field**		
**Total distance (cm)**	2599±813 (8)	2848±1036 (8)
**Center Time Male**	284±171 (4)	445±123 (4)
**Center Time Female**	546±101 (4)	464±190 (4)
**Rearing**	61±29 (8)	39±38 (8)
**Basal Body Temperature**		
**Female**	37.35±0.49 (4)	37.25±0.48 (4)
**Male**	36.8±0.09 (4)	36.8±0.28 (4)
**Stress-induced Hyperthermia**		
**Only males**	0.88±0.39 (4)	1.03±0.24 (4)
**Marble burying**		
**Males**	7.3±6.8 (6)	8.6±5.9 (8)
**Females**	7.7±6.7 (7)	6.25±7.3 (4)
**Inverted Screen**		
**Fell down (ratio)**	0/8 (0%)	2/8 (25%)
**Climbed up (ratio)**	7/8 (88%)	3/8 (38%)
**Hot Plate**		
**Latency to respond, sec**	10.2±4.8 (8)	11.35±5.4 (8)
**Acoustic Startle Response**		
**120 dB**	468±370 (8)	371±511 (8)
**PPI (%)**		
**pp4**	17.7±32 (7)	33.5±16 (5)
**pp8**	49±21 (7)	65±8.3 (5)
**pp12**	50±25 (7)	64±7.5 (5)
**pp20**	68±14 (7)	74±24 (5)
**Tail suspension**		
**Immobility time, sec**	224±58 (7)	186±107 (6)
**Fear conditioning**		
**Pre-CS freezing (%)**	4.0±6.5 (8)	12.9±14.9 (8)
**Post-CS cued freezing (%)**	34±29 (8)	35±27 (8)
**Formalin Paw (only males)**		
**Phase 1 flinches**	224±84 (2)	190±124 (6)
**Phase 2 flinches**	392±378 (2)	359±327 (6)

Data are expressed as mean ±SD (N). Statistical analysis was performed for all assays.

*- p<0.05.

#- p = 0.07–0.09 from WT cohort mates (unpaired t-test). Mice with startle amplitudes less than 100 were excluded from PPI analysis. Mice that climbed tails were excluded from tail suspension analysis.

**Figure 1:**
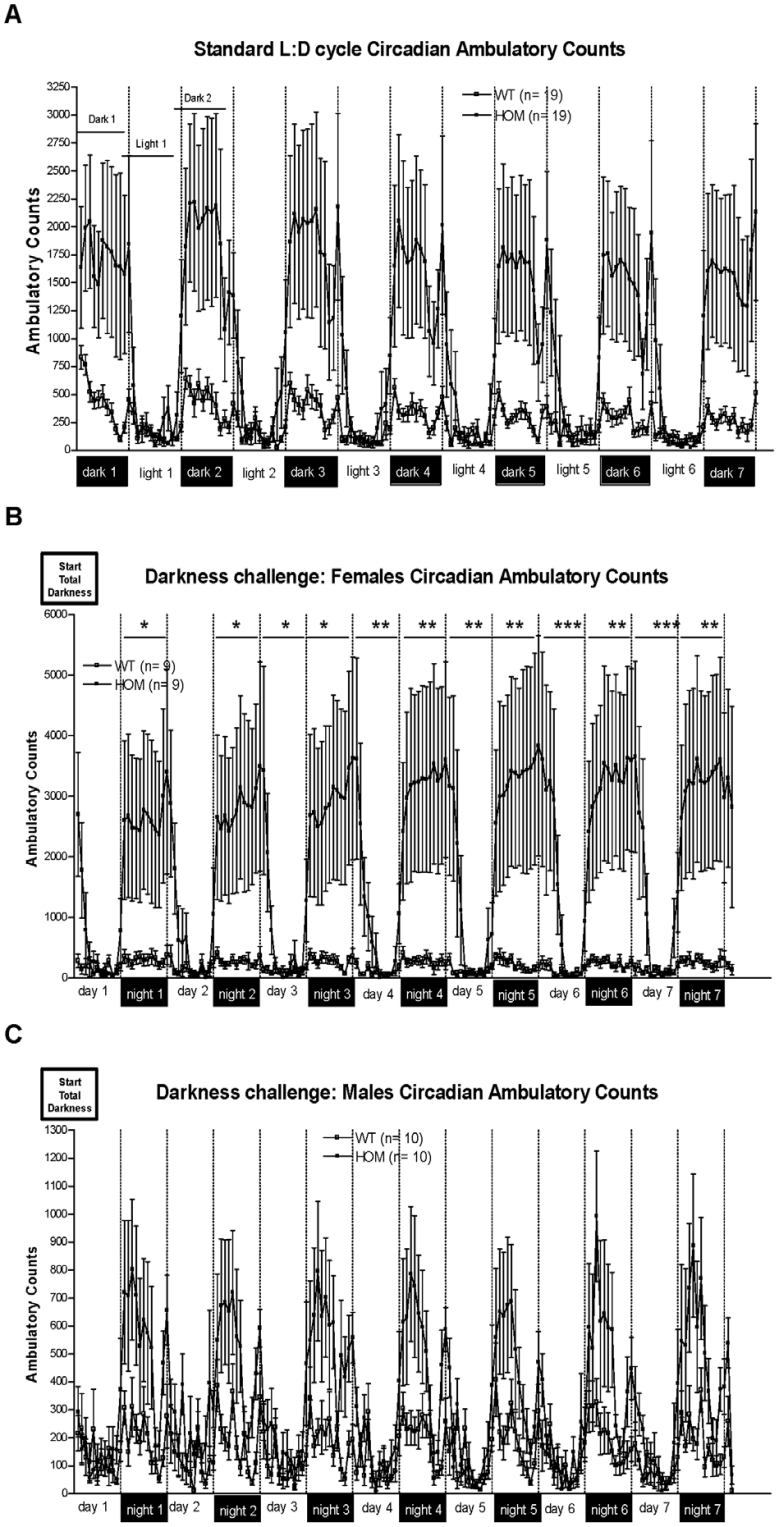
Tmub1 deletion resulted in increased ambulatory activity, but largely normal circadian rhythm in both light∶dark and dark∶dark protocols. A) Ambulatory counts during standard 12∶12 L∶D cycle. Data from male and female mice is combined on this graph since there was no significant effect of sex using RM ANOVA. B) and C) Ambulatory counts during continuous darkness challenge for females and males. *** - P<0.001, ** - P<0.01, * - P<0.05 (analysis of test period×sex×genotype interaction, Fisher LSD post-hoc following RM ANOVA).

Significant differences in locomotor activity were noted between WT and KO mice, with KO mice exhibiting higher activity during the dark phase (mouse subjective day) of the L∶D cycle in a home-cage environment. This was very obvious in the initial screening cohort and was seen in three additional cohorts created and tested over a two-year period. Therefore, expression of the phenotype was seen in multiple generations on the mixed background strains used to create these mice. In one of the larger cohorts, both males and females were monitored for circadian home-cage activity for 2 weeks; the first week consisted of exposure to a standard 12∶12 L∶D cycle (Figure 1A), and the second week continuous darkness (D∶D) (Figure 1B and 1C). There was a significant effect of genotype observed during the dark phase of a normal L∶D cycle with KO mice exhibiting greater activity [F(1,34) = 5.1, P<0.05]. There was no significant effect of genotype during the light phase. There was no significant effect of sex or interaction of sex with other measures for any part of the standard L∶D testing. The greater amount of locomotor activity during subjective day persisted into the D∶D challenge. During D∶D challenge a significant effect of genotype was observed during hours normally associated with darkness (subjective day) [F(1,34) = 5.2, P<0.05] and also a significant interaction of test period (repeated measure; RM)×sex×genotype [F(6,204) = 3.2, P<0.01]. Analysis of the interaction revealed that during the D∶D challenge a significant increase in activity during the normally dark period was observed in KO females. The increase in KO males was not significant. Interestingly, a significant genotype effect was also detected during the 12 hours normally associated with the light phase (subjective night) during the D∶D challenge [F(1,34) = 5.3, P<0.05]. During that time there was also a significant interaction of test day (RM)×sex×genotype [F(7,238) = 3.3, P<0.01]. As during the normally dark periods, the effect was significant only in females. Analysis of the interaction also showed that changes in subjective night-time activity during darkness challenge were observed starting on day 3 into the challenge. Visual inspection of the data found that increased activity in females continued on into the first couple of hours of the normally light phase (Figure 1B) before returning to expected light phase levels.

**Figure 2:**
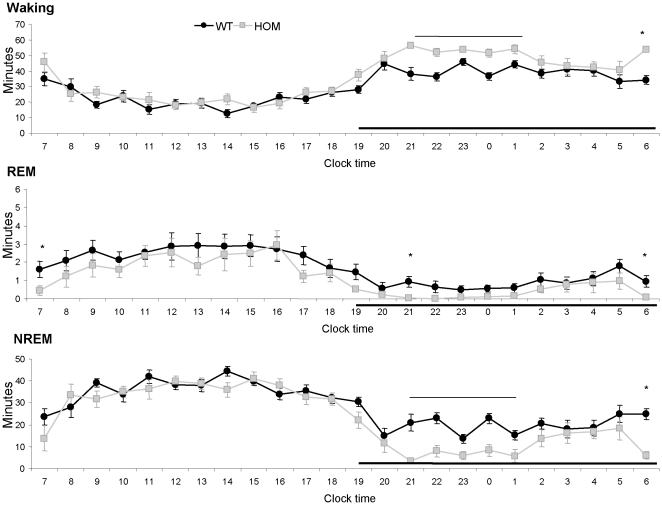
Tmub1 deletion resulted in enhanced waking and reduced REM and NREM sleep time during dark period. Mice were singly caged throughout the EEG/EMG recording in light cycle from 7 a.m. to 7 p.m. Dark phase is indicated by the black horizontal bars along the x-axis of clock time. Y-axis are minutes of waking, REM, or NREM sleep per one hour epoch. Data for KO and WT are shown in gray and in black respectively. Asterisks and horizontal lines above markers indicate significant difference between KO and WT group (p<0.05 in student t-test).

Telemetric recording of locomotor activity, EEG, EMG, and body temperature of mice in a home-cage environment provided additional support for the increased locomotor activity occurring only during subjective day (Figure S1A). The intensity of locomotor activity (Figure S1B) calculated as the activity counts per minute of wakefulness, was increased revealing an independence of increased activity from increased wakefulness. The total waking time of Tmub1 KO mice in the 12 hour dark phase was greater, to 125.6%, while NREM and REM sleep times were diminished, to 54.9% and 41.1%, respectively, compared to those of WT cohort-mates (table 2). In an hour by hour comparison, Tmub1 KO mice had significantly longer waking and shorter NREM times from the third through the seventh, and also the twelfth hour after the onset of darkness (Figure 2). In NREM, the EEG power density distribution in Tmub1 KO and WT mice was comparable, both in the light and dark phases (Figure S2A). During waking as well, the EEG power density distribution (the relative strength of specific EEG frequencies) was comparable in Tmub1 KO mice to that in WT mice in both the dark and light phases (Figure S2B). In the delta frequency range, a strong component of NREM sleep, although the averaged EEG power density in KO mice was numerically higher than that in WT mice, the difference was not significant overall or for any single hour.

**Table 2:** Tmub1 deletion resulted in enhanced waking and reduced NREM and REM time during dark period.

7am–7pm		WT (minutes)	KO (minutes)
(12 hr light period)	Waking	262.23±19.37	290.24±24.30
	REM	29.30±4.90	22.24±4.73
	NREM	428.47±17.15	407.52±24.49

7pm–7am		WT	KO
(12 hr dark period)	Waking	461.38±21.76	579.46±15.22 **
	REM	10.95±1.76	4.50±1.18**
	NREM	246.67±20.34	136.04±14.63**

Data presented as mean ± s.e.m.

**p<0.01 in student t-test in comparison to WT.

**Figure 3:**
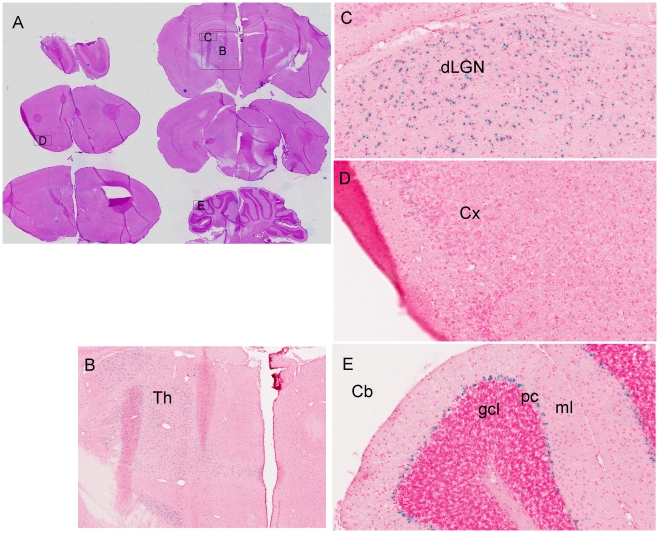
Tmub1 expression in adult brain was detected using B-Gal activity driven under the tmub1 promoter as described in Materials and Methods. A) Low magnification image of a series of thin brain section from KO (left side hemisphere) and WT (right side hemisphere) mice stained for B-Gal activity. LacZ expression was detected at low levels in the cortex (Cx) and higher levels in the thalamus (Th) and cerebellum (Cb). B), C), and D) Higher magnification of the thalamus, dorsal lateral geniculate nucleus (dLGN) in the Th, and Cx. E) High levels of LacZ in the Purkinje cell layer (pc) adjacent to the molecular layer (ml) and no detectable expression in the granule cells (gcl). (Original magnification: A, 0.7×; B, 5×; C, D, and E, 20×).

The expression of Tmub1 is reported to be high in the brain and the protein product is found in all parts of brain examined by western blot [14]. In situ hybridization of mouse brain reveals mRNA expression at P7 in the hippocampus, thalamus, cerebellum and cortex, and very low expression in adult brain (http://www.stjudebgem.org) [17]. Our LacZ expression (Figure 3) represents the activity of the endogenous Tmub1 promoter in the adult mice, and under standard exposure of thin sections of a series brain slices, expression of the mRNA is seen in the thalamus, cerebellum, and cortex (Figure 3A). Cellular density of LacZ reaction product was conspicuous in certain specific thalamic nuclei (Figure 3B) including the ventral posterior and, lateral geniculate nuclei (Figure 3C), and in the subthalamic nucleus and zona incerta. The LacZ signal was weak and only in a restricted set of cells in cortex (Figure 3D). In the cerebellum the blue LacZ reaction product was intense in Purkinje cells and a few basket/stellate cells (Figure 3E).

**Figure 4:**
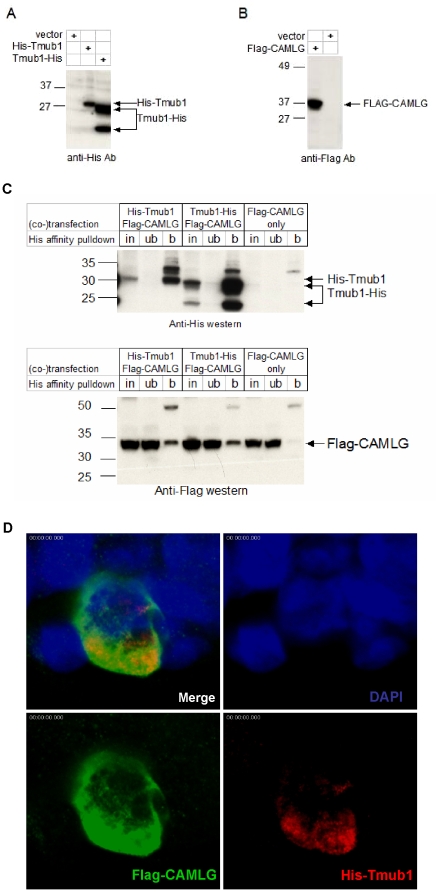
Interaction of Tmub1 and CAMLG in mammalian cells. A) His-Tmub1 and Tmub1-His were expressed in HEK293. Tmub1-His construct generated two products consistent to previous reports (Yang et al 2008; Della Fazia et al. 2005). B) FLAG-CAMLG was expressed HEK293. The molecular weight ladder is indicated as in kilodaltons. C) Tmub1 bound CAMLG when they were co-expressed in the HEK cells. Upper panel shows His-Tmub1 and Tmub1-His can be pulled down by anti-His affinity column; lower panel showed FLAG-Camlg was also pulled down only in the presence of Tmub1. in: input of lysate of transfected cells. Ub: fraction unbound to affinity column. b: bound to affinity column. D) Confocal images show Tmub1 and CAMLG were colocalized in HEK293 at cytoplasmic location. Green: anti-Flag for Flag-CAMLG; Red: anti-His for His-Tmub1, Blue: DAPI.

To discover the molecular function of Tmub1 we performed yeast two hybrid screening using full-length Tmub1 as a bait to identify proteins it binds specifically. Approximately 300 positive yeast clones were isolated from the Pretransformed Human Brain cDNA library. Of the seven proteins that hit more than twice, six were not in frame or corresponded to untranslated regions. Therefore, our attention was drawn to one that was present in more than 10% of all positive yeast clones characterized (33 clones). Its sequence revealed a full length, in-frame open reading frame encoding Calcium modulating cyclophilin ligand (CAMLG/CAML). The cDNA's encoding His-tagged Tmub1 and FLAG-tagged CAMLG were expressed in HEK293 (Figure 4A, B); co-immunoprecipitation experiments confirmed their interaction in these mammalian cells (Figure 4C).

To see whether Tmub1 and CAMLG proteins colocalized when expressed in HEK293 cells, immunostaining by anti-His tag and anti-FLAG tag was performed. The two proteins partially co-localized in the cytoplasmic region (Figure 4D).

## Discussion

The ubiquitin-like domain containing proteins are expected to have diverse roles in various biological processes. However, the studies of functions for many members in this family are only beginning. Tmub1 is one of a few UBL containing proteins that possess transmembrane domains. In this report deletion of Tmub1 in mice resulted in a robust, but very circumscribed, behavioral phenotype. Locomotor activity was greatly increased during subjective day (dark phase) in a home-cage environment. We did not observe this increased locomotor activity in other situations, such as a novel open field. The increased locomotor activity was concomitant with increased wakefulness as measured by EEG, but was not simply due to increased wakefulness because the rate of locomotor activity was also significantly increased. It is intriguing that the increased locomotor intensity, during the dark period and the very beginning of light period, did not continue during the remaining light period although a substantial amount of waking time still occurred. It will be interesting to determine how these two effects – increased intensity of locomotor activity and increased wakefulness – are related. Tmub1 KO mice were able to maintain a largely normal circadian rhythm of locomotor activity during a seven day complete darkness challenge, and continued to show the hyperactivity primarily during the normally dark periods. The female KO mice did extend the higher locomotor activity into the first couple of hours of the normally light period, but then returned to a low level appropriate for the light period. In the 24 hr EEG analysis, KO mice exhibited increased waking and decreased REM and NREM sleep during dark phase, while producing comparable amounts of waking and sleep during the light period. No abnormal EEG events were observed.

As Tmub1 is strongly expressed in the thalamus, a structure known to play roles in arousal and the expression of sleep and wakefulness [18], we suggest the behavioral phenotype is a result of altered neuronal activity in thalamus [19], [20]. The cerebellum, another area where Tmub1 is highly expressed, is not known for generation of sleep rhythms; however correlations between cerebellar gene expression with sleep and wakefulness have been reported [21].

Tmub1 has been previously shown to facilitate the recycling of GluR2 subunit of AMPA receptor to synaptic site [14]. The authors suggested that this process may be mediated by the interaction of GRIP and Tmub1/HOPS. They observed an interaction of Tmub1 and GRIP in HEK293 cells, but not in a yeast two-hybrid assay. It is possible that the interaction of Tmub1 with GluR2-GRIP is indirect and requires another mediator. In our yeast two-hybrid screen we identified a novel binding partner, CAMLG, for Tmub1. This was further confirmed by a co-immunoprecipitation pulldown assay. CAMLG was first identified as a cyclophilin B binding protein and shown to implicate cyclophilin B in the calcium signal transduction pathway in T cell activation [22]. A number of molecular interactors for CAMLG have since been identified [23]–[29]. CAMLG was found to be required for efficient EGF receptor recycling in studies from CAMLG wildtype and knockout primary fibroblasts [30]. CAMLG is also reported to be involved in recycling and endocytic processing of GABAA receptors [28]. In a cortical culture, using shRNA knockdown, this study revealed that the reduction of CAMLG translated into reduced GABAA receptors on the postsynaptic membrane and reduced GABA evoked current density and frequency of spontaneous IPSCs. The effect of CAMLG shRNA was specific to GABAA receptors since glutamate evoked current and spontaneous EPSCs remained unaltered in these neurons. The impact of GABAA receptor modulation, in sleep physiology, is widely accepted. It is interesting to speculate that Tmub1 and CAMLG proteins could work in concert to regulate cycling of receptors, such as GABA and glutamate receptors, to synapticmembranes in neuronal circuits critical for the control of locomotor activity and wakefulness in mice. Their modulation may prove to be interesting avenues for sleep research.

In conclusion our study reveals that Tmub1 directly binds to CAMLG protein and the physiological function of Tmub1 is in locomotor activity and wakefulness, likely via its molecular function in thalamic neuronal circuits.

## Materials and Methods

### Ethics Statement

All mice analyzed were maintained in an AAALAC-accredited animal facility at Lexicon Pharmaceuticals Inc. Mice were housed in a barrier facility at 22°C on a fixed 12-hour light and 12-hour dark cycle with free access to water and standard rodent chow (9F 5021; Purina, St Louis, MO). Procedures involving animals were conducted in conformity with the Institutional Animal Care and Use Committee guidelines that are in compliance with the state and federal laws and the standards outlined in the Guide for the Care and Use of Laboratory Animals. All experiments were approved by the Institutional Animal Care and Use Committee of Lexicon Pharmaceuticals, Inc.

### Knockout generation

**Figure 5:**
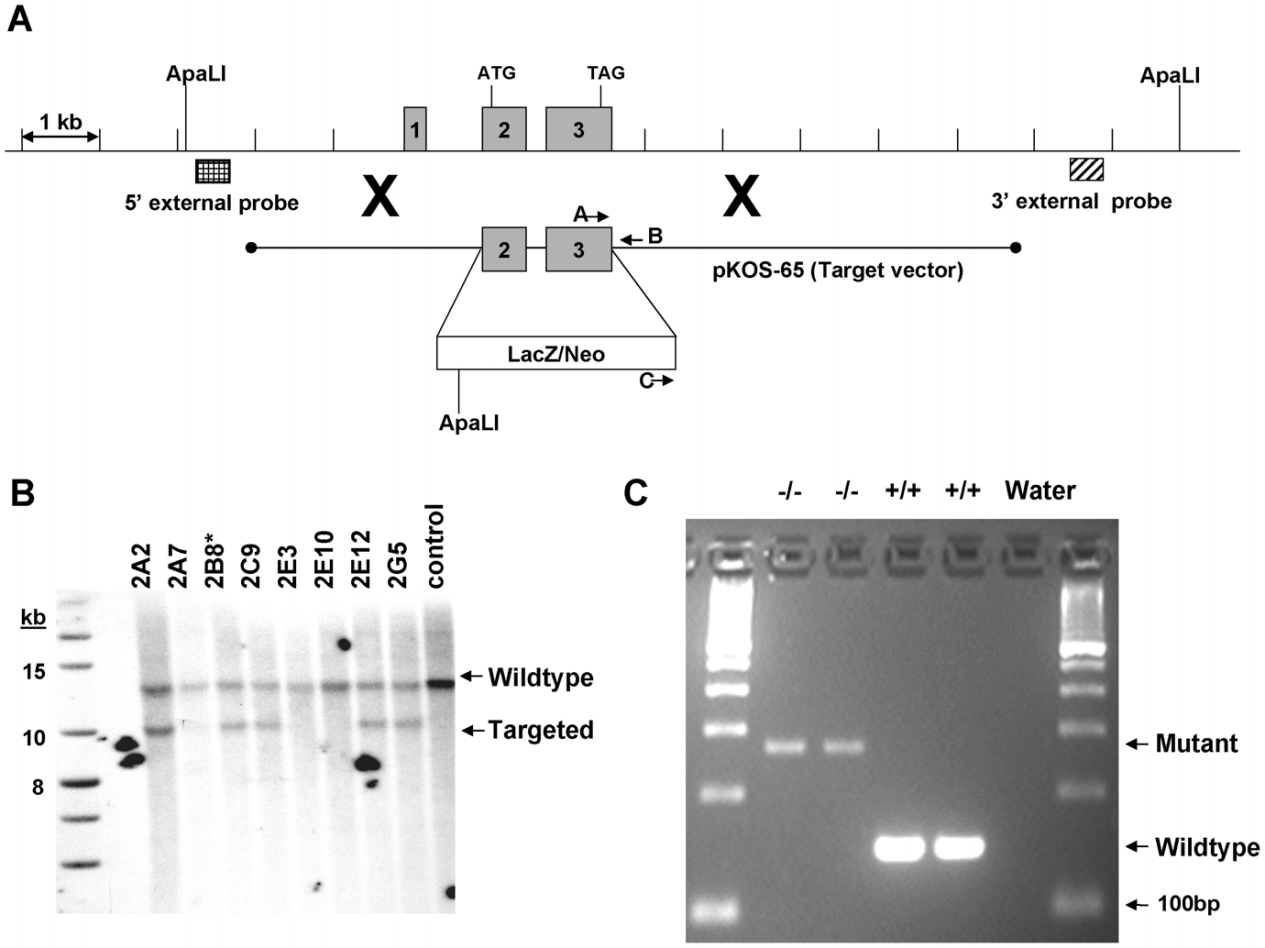
Knockout genetics. A) Construction of the Tmub1 gene targeting vector and Southern strategies for confirmation. A schematic representation of the Tmub1 locus is shown, including relevant ApaLI restriction sites used for confirmation Southern hybridization analysis. As described in the methods section, a genomic clone was isolated (pKOS-65) containing the exon 2–3 region to be deleted. A yeast-mediated homologous recombination (illustrated by large Xs) step first replaced the exon 2–3 region of the genomic clone with a yeast selection cassette. The final targeting vector was completed by replacing the yeast selection cassette with an ES selection cassette containing the LacZ reporter gene and a gene for G418 resistance. The 5′ and 3′ external probes used for Southern hybridization analysis were designed outside the target vector arms of homology and were generated by genomic PCR. Locations of the oligonucleotide primers used for genotyping animals are also noted. B) Southern hybridization demonstrating proper targeting of the ES cell clones. Genomic DNA was digested with ApaLI and hybridized with the 3′ external probe (see Method). Two correctly targeted clones were identified by Southern hybridization using the 5′ or 3′ external probes described in the text. Clones 2B8 and 2C9 were injected into blastocysts and clone 2B8 ultimately achieved germline transmission. C) Characterization of the knock out animals. Results of multiplex PCR described in the method show absent of wild type band and present of mutant band in two homozygous mice, while opposite results were seen in the wild type animals. Invitrogen's 100bp ladder was used as size marker.

The Tmub1 knockout line was generated by homologous recombination. The targeting vector was derived using the Lambda KOS system [31]. The Lambda KOS phage library, arrayed as 96 superpools, was screened by duplex-PCR using a primer pair upstream of exon 1 (Tmub1-1 [5′-CTCATAGGGACACAGTGCAGCAG] and Tmub1-2 [5′-GAGCATCTGTCACTCCTGACACC]) and a primer pair just downstream of exon 3 (Tmub1-8 [5′-GGTCACTCTCTCAGCTGGTCTTG] and Tmub1-9 [5′-CTAGGTCCTGAGTGCTCAAGCTAC]). Exons defined with reference to accession NM_022418.3. PCR-positive phage superpools were plated and screened by filter hybridization using the 410bp PCR product derived from primers Tmub1-5 [5′-GACTACAGGATGCTGCATCCTGG] and Tmub1-6 [5′-CTGTGTCAGGCTCTGGCTGTGC] as a probe. One genomic clone, pKOS-65, was isolated from the library and confirmed by sequence analysis. To generate the target vector, first gene-specific arms (5′- CTCTCTAGCTCCTTAGCACCTCTGGCAGCAGCACG-3′ and 5′- GCCTCCACGGGCTTTTGGCAGTGTAGCCAGCCCCTC-3′) were appended by PCR to a yeast selection cassette containing the URA3 marker. The yeast selection cassette and pKOS-65 were co-transformed into yeast and clones that had undergone homologous recombination to replace the 1005bp genomic region containing exons 2–3 with the yeast selectable marker were isolated. The yeast cassette was subsequently replaced with an ES cell selection cassette containing the reporter gene LacZ. The targeting vector was linearized by NotI digest and electroporated into 129/SvEv^Brd^ (Lex-1) ES cells (Figure 5A). G418/FIAU-resistant ES cell clones were isolated and correctly targeted clones were identified and confirmed by Southern analysis. A 509bp 5′external probe was generated outside the pKOS-65 arm of homology using PCR primers Tmub1-26 (5′-TTGAAGACCTGCTAGGGCTAC) and Tmub1-27 (5′-ATTATACCACACGGCAACTC). A 565bp 3′external probe was generated using PCR primers Tmub1-23 (5′-CTTAGCTAGGATGGCTCACG) and Tmub1-24 (5′-CCAACACAGCTTTGCTATCG). Southern analysis using the 26+27 5′probe detected a 12.8kb wildtype band and a 4.3kb targeted band in ApaLI digested genomic DNA while the 23+24 3′probe detected a 12.8kb wildtype band and a 10.1kb targeted band in ApaLI digested genomic DNA (Figure 5B). Two targeted ES cell clones were microinjected into C57BL/6 (albino) blastocysts. Resulting chimeras were mated to C57BL/6 (albino) females to generate mice that were heterozygous (Tmub1 +/−) for the exon 2–3 deletion mutation. Heterozygous mice were intercrossed, yielding Tmub1 −/− mice.

### Genotyping

Genomic DNAs were obtained from brain of wild type and homozygous animals. Gene specific primers pair (A: GCCCTTCTTTCCCCTGACCGCTACC and B: TGCGCCTTGGGGAATGAGGTCCAGA) together with neomycin specific and gene specific primer pair (C: GGCTGACCGCTTCCTCGTGCTTTAC and B) were employed to amplify wild type (+/+) and mutant (−/−) Tmub1 allele respectively in multiplex PCR reaction using High Fidelity polymerase (BioRad) according to the manufacture's instruction. The wild type band is 148bp while mutant band is 271bp (Figure 5C). PCR was carried out at initial denaturing step of 98°C for 2.5 min followed by 40 cycles of 98°C (15 sec), 69°C, (20 sec), 72°C (20 sec) then a final amplification step at 72°C for 1 minute. PCR products were subject to sequencing using 3730×l capillary sequencer (Applied Biosystems).

### Behavior assays

#### Subjects

Animals used for all studies were male (M) and female (F) knockout (KO) and wild type (WT) cohort mates bred in a mixed (C57BL/6J-*Tyr* ^c-Brd^×129S5/SvEvBrd) genetic background at Lexicon Pharmaceuticals. All mice were maintained under a standard light/dark cycle from 7 am to 7 pm. They were housed in groups of five in 30×20×20 cm acrylic cages with food and water freely available.

Mice were tested in a standardized battery of behavioral tests. A comprehensive phenotypic analysis included a wide range of behaviors as well as assays for cardiac, immune system, endocrine, and ophthalmic function. Cohort 1 (4 WT and 8 KO) was run through this standard phenotypic analysis battery routinely used at Lexicon Pharmaceuticals [16].

#### Circadian locomotor activity

Circadian locomotor activity was measured by a San Diego Instruments cage rack Photobeam Activity System (PAS-Home Cage, San Diego Instruments, San Diego, CA). Mice were individually housed starting at 5 pm on the first day of testing. The circadian chambers measured 48.2 cm×26.5 cm and allowed free access to food and water. For one week, animals were exposed to a regular 12-hour light/dark cycle (L∶D) with lights turning on at 7 am and turning off at 7 pm. Starting on day 7 the animals were monitored for an additional week in complete darkness (D∶D). Activity was recorded in one-hour intervals throughout testing.

#### Behavior data analysis

The Statistica 8.0 software package (StatSoft, Inc., Tulsa, OK) was used to determine significant differences between groups. Circadian activity data was analyzed using repeated measures ANOVA with sex and genotype as main effects and days across light cycle or days across dark cycle as repeated measure. Analysis of the same design was performed for continuous darkness challenge for periods normally associated with light cycle and periods normally associated with dark cycle. Where no significant effect of sex or sex×genotype interaction was determined the data from both sexes was analyzed together. Where significant interactions were determined Tukey HSD or LSD post-hoc analysis was performed to further analyze the differences between groups. Where no significant interactions were determined only the overall effects of genotype and/or sex are reported. The data from initial phenotypic evaluation was analyzed using unpaired two-tailed t-tests. The data is presented as Mean ± SEM. The lines on the dot-plot graphs represent group averages.

#### EEG/EMG/activity recording

A cohort of 19 naive male mice (10 months old, 9 KO and 10 WT littermates) were surgically implanted with F-20-EET biopotential transmitters (DSI, Data Science International, Wisconsin, USA) according to DSI EET device surgical manual for rodents. Briefly, mice were anesthetized with isoflurane. After the transmitter was placed on top of the intestines, biopotential leads were directed caudally to a dorsal neck incision through a subcutaneous tunnel. The animal was then placed in a stereotaxic apparatus for cranial immobilization. The skull was perforated through the bone but before the dura membrane at 2 mm either side of the sagittal suture and 1mm posterior to the bregma suture on left side and 1 mm anterior to the lambda suture on the right for ground and EEG leads implantation. EMG leads were secured in cervical trapezius muscles. Post-surgical analgesia (buprenorphine 0.05–0.1mg/kg) and replenishing fluid (saline 0.5 m)l were administered subcutaneously.

Following 10–14 days of recovery from surgery mice were transferred into the EEG lab and acclimated for 2 days before experiment. After acclimation EEG, EMG, body temperature, and locomotor activity data were continuously acquired for 24 hours with DataquestART 4.0 (Data Sciences International) data acquisition system. EEG and EMG signals were amplified and filtered at 0.3–30 Hz and 20–200 Hz respectively, digitized at a sampling rate of 250 Hz. Locomotor activity data were collected as relative animal movement counts per minute. Acquired EEG/EMG data were visually inspected for a successful recording then autoscored with SleepSign for Animal software (Kissei Comtec Co. Japan) into three different vigilance states: NREM, REM or Waking. Averaged EEG power spectral was computed from all 10s epochs scored into the same vigilance state during the 12 hour light or dark phase. EEG power density was calculated from the power spectral at each frequency as percentage of sum of all frequency.

### LacZ histochemistry

LacZ gene as a reporter is within the targeting cassette. The LacZ gene expression represents the activity of the endogenous Tmub1 promoter. The LacZ expression was measured by 5-bromo-4-chloro-3-indolyl-D galactopyranoside (x-gal) reaction on 4µm brain slices of knockout and wild type mice as described by Vogel et al [32].

### Immunohistochemistry

Four percent paraformaldehyde fixed, paraffin-embedded tissue sections at 4 µm were mounted on positively charged glass slides (Superfrost Plus, Fisher Scientific, Pittsburgh, PA) and first deparaffinized in xylene and re-hydrated in PBS before incubating in the primary antibody. Endogenous peroxidase activity was blocked by incubation in 3.0% hydrogen peroxide in PBS for 5 min. Tissue sections were then blocked with 20% normal horse serum for 10 minutes and then primary antibody mouse anti-GAD67 (MAB5406; Chemicon (Millipore) at 1∶500 was applied for 1 hour at 37 degrees centigrade. After rinsing, sections were incubated for one hour in a 1∶500 dilution of the biotinylated secondary antibody (Horse anti-Mouse IgG, Vector, Burlingame, CA). Bound antibodies were detected by an avidin-biotin complex method (Elite ABC Kit, Vector) with 3,3′-diaminobenzidine as chromogenic substrate following the manufacturer's instructions. Negative controls included substitution of non-immune serum for the primary antibody and omission of the primary antibody. Sections were mounted with Permount (Fisher Scientific, Pittsburgh, PA).

### Yeast Two-hybrid screening

Tmub1 full-length cDNA was cloned into yeast expression vector pGBKT7. The pGBKT7-Tmub1 plasmid was transformed into yeast strain AH109. The Yeast Two-hybrid screening was carried out according to the manufacturer's protocol (Clontech) using Pretransformed Human Brain MATCHMAKER cDNA library (Clontech). The Human cDNA inserts were cloned into yeast expression vector pACT2. There are 5×10^6^ independent clones in this library and approximately 8.2×10^6^ clones were screened. Positive clones were selected on plates supplemented with X-α-Gal lacking leucine, tryptophan, and histidine. Positive clones were used as templates, and cDNA fragments of the proteins that bind to Tmub1 were amplified by PCR using primers flanking cDNA inserts in vector pACT2.

### Biochemistry

Tmub1 was cloned into pIRES-puro2 expression vector with 6×His at N-terminus or C-terminus. CAMLG was also cloned into pIRESpuro2 vector with Flag tag at N-terminus. The constructs were transiently transfected to HEK293 cells and expression of recombinant proteins was confirmed by western blot using anti-His (Qiagen) and anti-Flag (Sigma) antibodies. Cells were extracted in lysis buffer (20mM Tris pH8.0, 150mM NaCl, 1% Triton X-100, 1mM EDTA, 1× protease inhibitor cocktail) to obtain cell lysate. The coimmunoprecipitation was carried out using anti-His or anti-Flag antibody in cell lysate and the anti-Flag or anti-His antibodies were used to confirm the presence of Tmub1 and CAMLG in the immunoprecipitates.

## Supporting Information

Figure S1 Deletion of Tmub1 resulted in increased locomotor activity. Mice were singly caged throughout the EEG/EMG/BT/activity recording with light cycle from 7 a.m. to 7 p.m. Dark phase is indicated by the black horizontal bars along the x-axis of clock time. Data for KO and WT are shown in gray and in black respectively. A) Tmub1 deletion resulted in dramatic increase in locomotor activity during dark phase. Y-axis represents the averaged activity counts in the same genotype group for each hour. B) Tmub1 deletion resulted in significant increase in locomotor activity intensity during dark phase. Locomotor activity intensity was calculated as activity counts per waking minutes. Y-axis represents the averaged activity counts per waking minutes in the same genotype group for each hour.
(0.19 MB TIF)

Figure S2 Deletion of Tmub1 did not affect EEG power density distribution across different frequency bands in NREM (A) and waking (B) state. EEG power density is computed from averaged 2-hour FFT (fast Fourier transform) and expressed as percentage of spectral power in delta, theta, alpha, beta, and gamma frequency over total spectral power in NREM state. Data for KO and WT are shown in gray and in black respectively. Mice were singly caged throughout the EEG/EMG/BT/activity recording with light cycle from 7 a.m. to 7 p.m. Dark phase is indicated by the black horizontal bars along the x-axis of clock time.
(0.23 MB TIF)
