# Supplementary figures and images for: Transmembrane and Ubiquitin-Like Domain Containing 1 (Tmub1) Regulates Locomotor Activity and Wakefulness in Mice and Interacts with CAMLG
Source: PLoS One. 2010 Jun 22;5(6):e11261. doi: 10.1371/journal.pone.0011261 (PMC2889838; doi:10.1371/journal.pone.0011261)

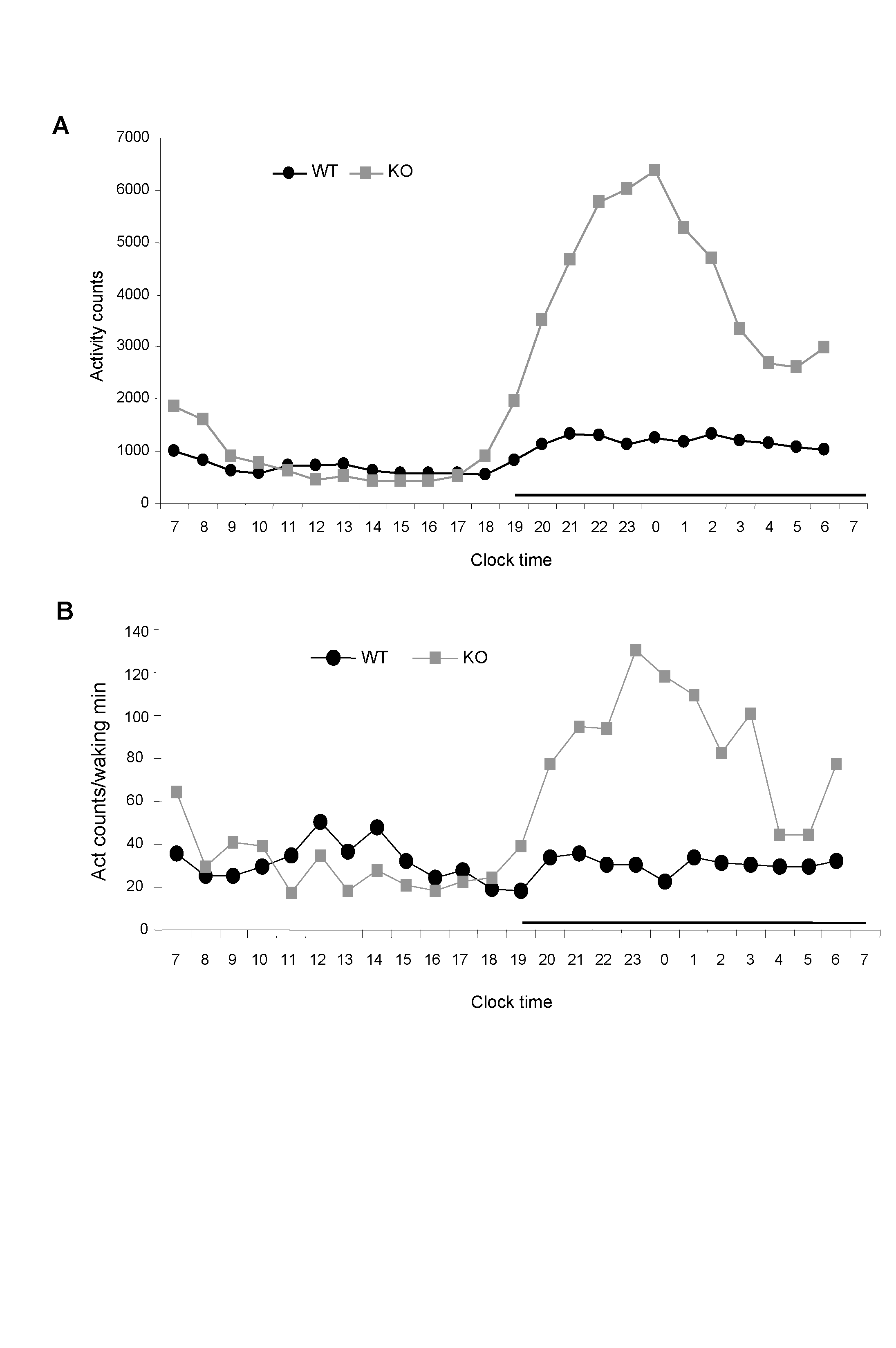

Supplement: Figure S1 — Deletion of Tmub1 resulted in increased locomotor activity. Mice were singly caged throughout the EEG/EMG/BT/activity recording with light cycle from 7 a.m. to 7 p.m. Dark phase is indicated by the black horizontal bars along the x-axis of clock time. Data for KO and WT are shown in gray and in black respectively. A) Tmub1 deletion resulted in dramatic increase in locomotor activity during dark phase. Y-axis represents the averaged activity counts in the same genotype group for each hour. B) Tmub1 deletion resulted in significant increase in locomotor activity intensity during dark phase. Locomotor activity intensity was calculated as activity counts per waking minutes. Y-axis represents the averaged activity counts per waking minutes in the same genotype group for each hour. (0.19 MB TIF) [file pone.0011261.s001.tif]

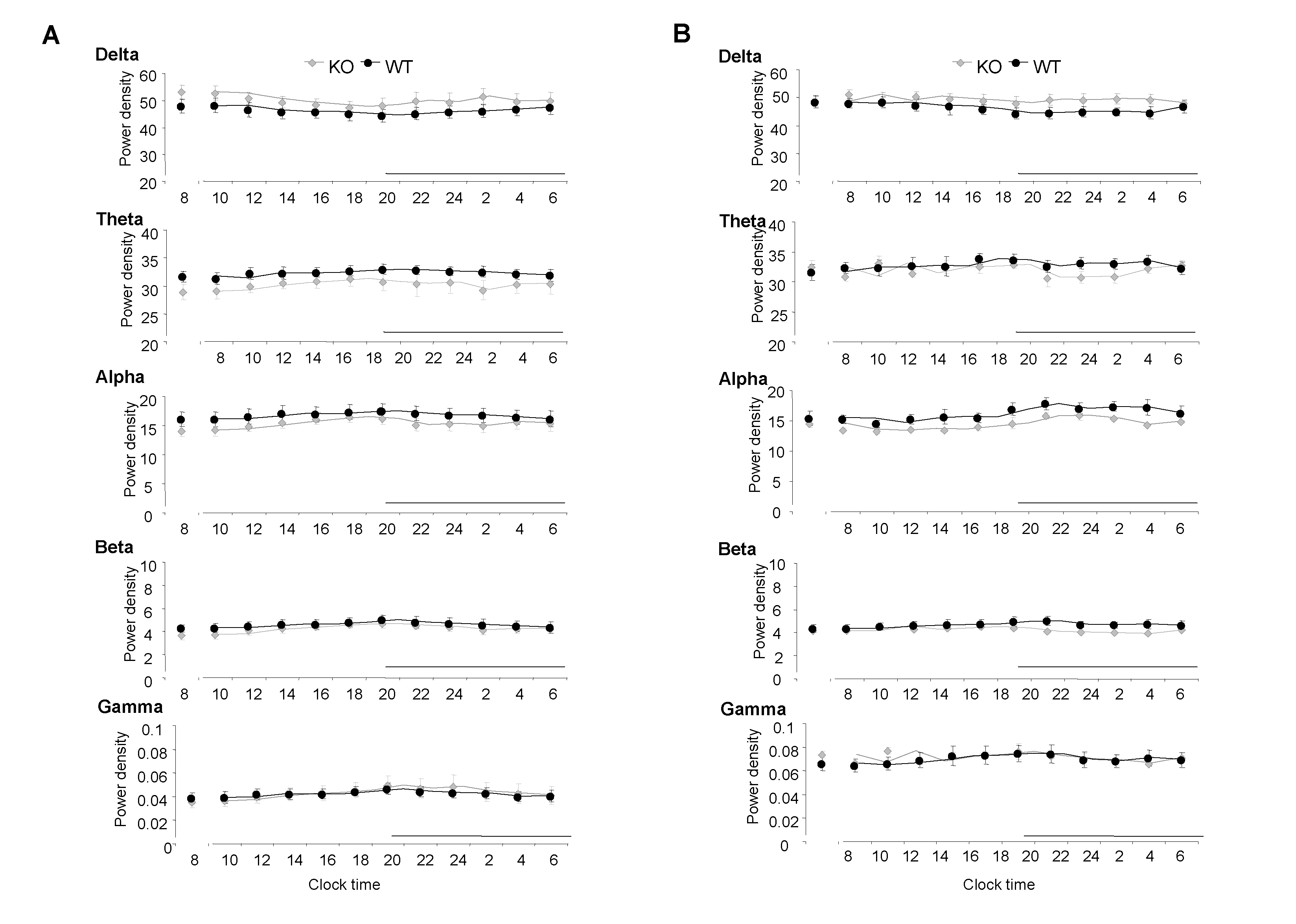

Supplement: Figure S2 — Deletion of Tmub1 did not affect EEG power density distribution across different frequency bands in NREM (A) and waking (B) state. EEG power density is computed from averaged 2-hour FFT (fast Fourier transform) and expressed as percentage of spectral power in delta, theta, alpha, beta, and gamma frequency over total spectral power in NREM state. Data for KO and WT are shown in gray and in black respectively. Mice were singly caged throughout the EEG/EMG/BT/activity recording with light cycle from 7 a.m. to 7 p.m. Dark phase is indicated by the black horizontal bars along the x-axis of clock time. (0.23 MB TIF) [file pone.0011261.s002.tif]
